# Dementia family carers’ needs and wants for technological solutions to their work–care reconciliation challenges: semi-structured interviews

**DOI:** 10.1007/s10354-026-01136-9

**Published:** 2026-02-27

**Authors:** Alice Spann, Marieke Spreeuwenberg, Mark Hawley, Luc de Witte

**Affiliations:** 1 Center for Public Health, Medical University of Vienna, Kinderspitalgasse 15, 1090 Vienna, Austria; 2 Faculty of Health Medicine and Life Sciences, Maastricht University, Minderbroedersberg 4-6, 6211 LH Maastricht, The Netherlands; 3 Faculty of Medicine, Dentistry and Health, University of Sheffield, 217 Portobello, Broomhall, S1 4DP Sheffield, UK; 4 Centre of Expertise Health Innovation, The Hague University of Applied Sciences, Johanna Westerdijkplein 75, 2521 EN The Hague, The Netherlands

**Keywords:** Working carers, Technology, Challenges, Dementia, Interviews, Wants and needs, Berufstätige pflegende An- und Zugehörige, Technologie, Herausforderungen, Demenz, Interviews, Wünsche und Bedürfnisse

## Abstract

Most people with dementia who live in the community are cared for by family, friends, or neighbors. Many of these unpaid dementia carers have to balance caring with paid work, which can present them with several challenges. Technology can offer potential solutions, independent of an already strained social care system. This qualitative study aimed to explore working dementia carers’ needs and wants regarding technological solutions for their work–care reconciliation challenges. We conducted semi-structured interviews with 16 (10 women, 6 men) working carers of community-dwelling people with dementia in Scotland. Data were analyzed thematically to identify key themes. Carers wanted solutions for seven main issues: (a) care management; (b) attending appointments; (c) entertainment and companionship for the person with dementia; (d) dealing with psychological and psychosocial stress; (e) safety concerns; (f) accessing information, and (g) personal care. The technological solutions most carers had experienced focused on care management, safety concerns, and access to information. Few if any carers had experience with technologies for entertainment and companionship for the person with dementia, their own psychological and psychosocial well-being, attending appointments, and personal care. Some carers made suggestions for technologies they were not aware already existed, highlighting the need for effective signposting to technological solutions tailored to their individual needs. Our findings are relevant for healthcare professionals, organizations, and employers seeking to support working carers.

## Abstract (de)

Die meisten Menschen mit Demenz, die in ihrer häuslichen Umgebung leben, werden von Familienangehörigen, Freunden oder Nachbarn gepflegt. Viele dieser pflegenden An- und Zugehörigen müssen die Pflege mit ihrer Erwerbsarbeit vereinbaren, was sie vor verschiedene Herausforderungen stellen kann. Technologie kann potenzielle Lösungen bieten, unabhängig von einem bereits überlasteten Sozialsystem. Diese qualitative Studie hatte zum Ziel, die Bedürfnisse und Wünsche berufstätiger pflegender An- und Zugehöriger von Menschen mit Demenz hinsichtlich technologischer Lösungen für ihre Herausforderungen bei der Vereinbarkeit von Beruf und Pflege zu untersuchen. Wir führten halbstrukturierte Interviews mit 16 (10 Frauen, 6 Männer) berufstätigen pflegenden An- und Zugehörigen von Menschen mit Demenz durch, die in Schottland in ihrer häuslichen Umgebung leben. Die Daten wurden thematisch analysiert, um Schlüsselthemen zu identifizieren. Die pflegenden An- und Zugehörigen wünschten sich Lösungen für sieben Hauptprobleme: (a) Pflegemanagement, (b) Wahrnehmung von Terminen, (c) Unterhaltung und Gesellschaft für Menschen mit Demenz, (d) Umgang mit psychischem und psychosozialem Stress, (e) Sicherheitsbedenken, (f) Zugang zu Informationen und (g) Körperpflege. Die technologischen Lösungen, mit denen die meisten pflegenden An- und Zugehörigen Erfahrung hatten, zielten auf Pflegemanagement, Sicherheitsbedenken und Zugang zu Informationen ab. Nur wenige pflegende An- und Zugehörige hatten Erfahrung mit Technologien für Unterhaltung und Gesellschaft von Menschen mit Demenz, ihr eigenes psychisches und psychosoziales Wohlbefinden, die Wahrnehmung von Terminen und Körperpflege. Einige pflegende An- und Zugehörige schlugen Technologien vor, von denen sie nicht wussten, dass sie bereits existierten, was die Notwendigkeit einer effektiven Aufklärung über technologische Lösungen für ihre individuellen Bedürfnisse unterstreicht. Unsere Ergebnisse sind relevant für Beschäftigte im Gesundheitswesen, Organisationen und Arbeitgeber, die berufstätige Pflegepersonen unterstützen möchten.

## Background

Dementia refers to degenerative brain processes that are generally progressive and will affect 75 million people worldwide by 2030. Symptoms include emotional, psychological, cognitive, and behavioral issues and can undermine independent living [64]. Most people with dementia live in their communities supported by unpaid family carers, i.e., family, friends, or neighbors [1, 48]. Roughly 4.9 million people in the United Kingdom combine unpaid care with paid work, which amounts to 1 in 7 people in paid work [20]. This number increased during the Covid-19 pandemic [21, 45]. Carers in full-time work are most likely to care for people with dementia [18]. Although precise figures are unavailable, the Centers for Disease Control and Prevention estimates that about half of unpaid dementia carers in the United States balance caregiving with paid work [22]. These numbers are likely to rise due to the projected increases in dementia prevalence, pressures on health and social care provision linked to funding constraints and workforce shortages, and rising retirement ages [11, 12, 15, 23, 47–49, 59, 64, 65].

Care needs may include personal care, instrumental activities of daily living, social and emotional support, and safety monitoring, typically fluctuating and intensifying as dementia progresses [26]. Because of this complexity and unpredictability, caring for people with dementia is often more demanding than caring for someone with other conditions [38, 44], requiring more care overall and emotional labor due to slow decline, interpersonal conflict, and constant vigilance for behavioral and psychological symptoms. Accordingly, dementia carers experience higher stress, poorer physical and mental health, and reduced quality of life compared to other carers [19, 29, 34, 36, 40]. Nonetheless, caregiving can also provide meaning, reciprocity, and a sense of purpose [9, 25, 28]. Paid work may similarly offer achievement, social contacts, respite, and financial security [8, 14, 54], yet balancing both work and care can create significant challenges for carers with adverse consequences for health, relationships, and employment when support is insufficient [52, 56–58, 62, 65].

In an under-resourced social care system [23, 59], technology can offer carers urgently needed solutions for their work–care reconciliation challenges. Here, technology is defined as any electronic and/or digital device or application. While technologies for carers [7, 50, 53, 60] and people with dementia [3, 4, 37, 66] are increasingly investigated, little attention has yet been given to technology that can help carers of people with dementia to better combine work and care. A recent review identified 16 publications, academic and grey, addressing technological solutions for working carers [51]—covering Web-based technologies, technologies for direct communication, monitoring technologies, and task-sharing tools—used by carers to have peace of mind; manage their care network; stay connected to their workplace when working from home; decrease the care demand; access information; improve their mental health, resilience, and relationship with the cared-for person; and save time and money. However, none took a bottom-up approach to explore which challenges working carers themselves wanted technology to address. Given that many carers are not aware of available technologies and where they can procure them [17], UK signposting platforms such as alzproducts.co.uk, liftedcare.com, livingmadeeasy.org.uk, and meetadam.org represent welcome developments. However, while most of these sites enable carers to filter technologies for specific needs, none of them yet include filters specific to the challenges carers face when combining work and care for people with dementia. To address this gap, this paper explores which work–care reconciliation challenges working dementia carers want, need, and use technological solutions for. It does not attempt to catalogue available technologies beyond mentioning those explicitly referenced by participants.

## Methods

For this qualitative study, we conducted semi-structured interviews that allow researchers to gain a first-hand understanding of people’s lived experiences and views [5, 13]. We used Witzel’s [63] problem-centered approach, which employs a topic guide to prompt interviewees’ narration on central issues, thus facilitating the in-depth exploration of the issues while ensuring that essential themes are addressed. In addition, a brief questionnaire was administered to capture interviewees’ contextual demographic data.

The topic guide used deliberately broad questions that invited carers to describe the most difficult aspects of combining work and care, how caring affected their work, and how work affected their caring. These questions were prioritized over directly asking which technologies they already used or wanted to use (although those issues were also discussed) as we wanted to avoid carers’ responses to be restricted by their current understanding and knowledge of technology (which, according to Carers UK, [17], is often limited—not least by the rapid advances in technology research and development). To that effect we also asked carers what they would want technology to do for them if there were no limitations. A pilot interview confirmed the topic guide’s suitability. Interviews were conducted in Scotland between March and July 2019 at a place most convenient for interviewees: nine chose their home; five chose neutral places like pubs, cafés, or meeting rooms; and one requested a phone interview. Interviews lasted between 35 and 120 min (mean: 90 min). All interviews were audio-recorded and observational notes were taken during and after.

### Ethical approval

Full ethical approval was granted by the ScHARR Research Ethics Committee at the University of Sheffield (Reference 022994). All participants were fully informed about the study, including their right to withdraw at any point without consequence, and gave their explicit consent before the interview started. Confidentiality was ensured throughout the study and data were stored securely. All participants received a pseudonym to ensure their anonymity.

### Participants

We used a purposive sampling strategy [5] to ensure a high level of generalizability regarding age, gender, and participants’ levels of autonomy at work (defined as control over workhours, workplace, and breaktimes) that we reasoned would impact their ability to manage care-related issues and use technology when working. Carers were eligible if they (a) were in paid work for at least 20 h/week and (b) provided care for a person with dementia for at least 5 h/week. They also (c) had to have been working carers for at least 6 months, and (d) care for a person with dementia living outside of residential care settings in Scotland.

### Recruitment

We used a multipronged recruitment approach. Carer organizations, the chamber of commerce, trade unions, the researchers’ professional network, and a random selection of businesses operating in Scotland were asked to distribute our recruitment flyers among their employees, clients, and contacts and to post an advertisement for the study on their social media channels. Flyers were also pinned to several community notice boards, handed out at dementia support groups, and placed at dementia day-care centers. Carers were invited to contact the research team if they fit the eligibility criteria and wanted to be involved. One carer organization forwarded the contact details of interested clients with their consent, who were then contacted by the research team. We also asked participants to forward our recruitment flyer to other carers they knew who fit the inclusion criteria.

### Analysis

Interviews were transcribed and read multiple times to ensure familiarity with the texts. The data were analyzed using the thematic analysis approach described by Braun and Clarke [10]. We used an inductive, iterative, descriptive process to identify themes. The research aims were used to guide the analysis and to discover relevant themes. We first coded the initial five transcripts using NVivo 12 software. Codes were then revised and used to code the remaining transcripts. Codes were added and revised throughout the process and ultimately combined into clusters of meaning to form the themes presented hereafter. Emerging themes were discussed within the research team and other work–care scholars. Appendix Table A1 provides an overview of the themes, subthemes, and sample quotes. Each interviewee was then sent a short summary of their interview, using the themes that emerged from our analysis. Interviewees were asked to confirm whether this interpretation was true to their experience and views, and whether they wanted anything added, clarified, or amended. Six interviewees (37.5%) responded. Any clarifications or additional information they provided was included in the ongoing analysis.

## Findings

In total, 16 working carers were interviewed (ten women, six men), aged 27–70 years (mean: 50.6). Seven carers were employed full time, six were self-employed, one was partially retired, and two were small business owners in addition to having full-time employment. Reported care hours ranged from 5 to, in one case, more than 75 h per week. Many carers found it difficult to estimate weekly care hours, since much of their caring was unplanned and not always recognized as care (e.g., shopping or home maintenance). The carers supported 17 people with dementia: 13 were parents (including one carer supporting both parents), one a parent-in-law, one an uncle, and two a spouse. Three carers had experienced major changes shortly before the interview: two had reduced from full-time work to fewer than 20 h/week as self-employed carers, and one had begun transitioning into retirement following the death of the person with dementia. In line with the study’s eligibility criteria, only carers’ circumstances prior to these changes were included in the analysis. Table 1 summarizes participants’ characteristics.

**Table 1:** Participant characteristics

*Carer*		*Women (N = 10)*	*Men (N = 6)*	*Total n (N = 16)*
*Age*	< 40	1	3	*4*
	40–60	7	1	*8*
	> 60	2	2	*4*
*Employment status*	Employed	6	3	*9*
	Self-employed	2	2	*4*
	Employed and self-employed	1	1	*2*
	Partially retired, employed	1	0	*1*
*Weekly work hours*	< 35	1	1	*2*
	35–40	7	2	*9*
	> 40	2	3	*5*
*Duration of care (years)*	0.5–2	3	2	*5*
	3–6	2	3	*5*
	> 6	5	1	*6*
*Weekly care hours*	< 10	2	2	*4*
	10–20	4	1	*5*
	21–40	3	2	*5*
	> 40	1	1	*2*
*People with dementia*				*Total n (N* *=* *17)*
*Relationship to carer*	Parent	–	–	*13*
	Parent-in-law	–	–	*1*
	Other parental generation	–	–	*1*
	(Ex‑)Spouse	–	–	*2*
*Age (years)*	< 70	–	–	*4*
	70–80	–	–	*3*
	> 80	–	–	*10*
*Dementia diagnosis*	Alzheimer’s	–	–	*5*
	Vascular	–	–	*3*
	Fronto-temporal	–	–	*2*
	No official diagnosis	–	–	*1*
	*N*/A	–	–	*6*
*Dementia stage *(CDR) ^a^	Moderate	–	–	*5*
	Moderate–severe	–	–	*4*
	Severe	–	–	*8*

^a^*CDR* Clinical Dementia Rating Scale, staging based on participants’ descriptions [55]

Our analysis produced seven themes representing challenges carers wanted solutions for. It is important to note that, due to the nature of our research focus, technological solutions might not be immediately evident for some of the challenges identified. These challenges were (a) care management; (b) attending appointments; (c) entertainment and companionship; (d) psychological and psychosocial stress; (e) safety concerns; (f) accessing information, and (g) personal care. The order in which these challenges are presented does not constitute any prioritization. Not every carer^1^ described experiencing every challenge, but all of them reported experiencing or having experienced more than one. Many carers we interviewed did not experience their work–care reconciliation efforts as static due to fluctuating care needs and changing circumstances at work (e.g., getting a new line manager, business trips, meeting deadlines). Accordingly, the challenges they experienced could change and with them their priorities for support. Appendix Table A1 provides an overview of the identified work–care reconciliation challenges carers wanted solutions for, along with sample quotes. Additional quotes can be found in Appendix B to add authenticity and create an emotional connection to carers’ lived experience. The following text signposts to these quotes via (Q_NO_).

### Care management

#### Coordinating the care network

Care networks included unpaid (i.e., family members, friends, neighbors) and sometimes paid (i.e., live-in care workers, personal assistants, and assisted living facility manager) members. Carers needed to coordinate responsibilities with their care network and exchange information about the condition, habits, mood, or upcoming appointments of people with dementia. Some arrangements with care network members were stable and reliable, requiring little managerial effort. Other carers had to rely on family members who had considerable care needs themselves or manage their care arrangement on a day-by-day basis, which could cost a lot of time and effort and lead to work disruptions. Carers used their phone to text, call, or email their network. Many carers appreciated instant messenger apps for allowing them to communicate with their whole network at once; exchange documents, images, and videos; and store records of their exchanges. Some carers wanted reassurance that a member of their care network had been to check in with the person with dementia to make sure they were alright (see also safety concerns). However, one carer found that some network members did not want or know how to use even user-friendly technology such as texting and wondered whether there was yet simpler technology that could notify them if someone had visited to check on the person with dementia.

#### Coordinating care providers

Some carers felt that the care provided by services was sometimes inadequate and that their instructions were not followed. Maggie, who works from home, described her frustration over having to interrupt her work to help her mother’s home care workers (Q1). Registering their complaints (e.g., via phone or email) and continuously having to explain what they wanted care workers to do could be time-intensive and cause conflict. Theresa wanted remote access to an online version of care workers’ logbook reports, which they needed to complete after each care visit, and which were kept in hardcopy form in the home of the person with dementia (Q2). Max, who used his lunch break to head home and help his mother eat her lunch, was frustrated that he had no way of knowing when care workers would arrive, making him wait for them to finish their tasks before he could help his mother and get back to work. He suggested that technology could inform him when exactly care workers would arrive so he could make his arrangements accordingly. Carers who privately hired care workers suddenly found themselves as employers with all the associated responsibilities. This required substantial administrative effort and could be overwhelming for carers with no experience managing employees (Q3).

### Attending appointments

#### Attending medical and similar appointments

Conflicting work hours of health- and social care professionals (HCPs) were challenging. Medical or similar appointments made well in advance presented relatively few problems, as carers could either arrange for time off work or for their care network to accompany the person with dementia. Some appointments, however, could be very short notice. To attend, many carers depended on the goodwill of their line managers or on colleagues taking over for them. Sometimes work had to be prioritized, e.g., when they had important meetings (Q4). Attendance on short notice was also not possible when the distance to the carer’s workplace was too great.

#### Attending business meetings

Many carers used videoconferencing software for business meetings when working remotely. The chat functions were particularly appreciated, as they allowed carers to unobtrusively communicate if they needed to leave for care-related reasons.

#### Arranging medical or similar appointments

Arranging appointments with HCPs was challenging for carers, as the conflicting hours meant they needed to find time to do this while at work. Emails were thus preferred over phone calls, as they were easier to accommodate (Q5).

### Entertainment and companionship

#### Providing entertainment and companionship

The continuous cognitive decline and social withdrawal of people with dementia were big concerns for carers. Many, particularly carers who worked and cared simultaneously, felt guilty for not being able to keep the person with dementia more company (see also psychological and psychosocial stress). One carer felt that entertainment like TV or radio was not stimulating enough, while also musing that the person with dementia could find it difficult to learn using new technology. Another remembered the positive effect a small dog had had on his wife in terms of providing companionship and wondered whether technology could have a similar effect.

#### Enabling active participation in society

Some carers wanted the person with dementia to actively participate in society. Carers suggested a website that could collate information on dementia-friendly events in the neighborhood, alongside self-driving cars people with dementia could use to get around.

#### Enabling people with dementia to communicate

Dementia massively impacted some peoples’ ability to communicate. Some could no longer give coherent responses, while others appeared to have lost the ability to speak altogether. Their carers expressed a desire for technology that could facilitate communication (Q6).

### Psychological and psychosocial stress

Carers reported having to deal with complicated emotions. A prominent emotion was guilt, e.g., for having to prioritize work over caring and, vice versa, for not being able to give their family as much attention as needed, or for considering moving the person with dementia to a care home as they felt they could no longer cope. While some carers managed to find positive aspects to their situation, others felt overwhelmed or alone. Some reported having conflicts with siblings who refused to become more involved with caring for their parents. Almost all talked about having to make personal sacrifices they sometimes felt sad or resentful about. Relationships with romantic partners, friends, and family often suffered and some carers felt abandoned or not understood by their social network. Role reversal and dementia-related personality changes were often difficult to accept, as was getting to terms with slowly losing their loved one. Dementia could sometimes exacerbate already difficult relationships between the carer and the person with dementia. Refusal of help (in the form of, e.g., home care services, day-care-centers, technology) could be a great source of conflict and frustration. Emotional labor and constantly being ready to spring into action were very exhausting for carers, with many already experiencing adverse health effects caused by exhaustion. Accordingly, some carers made a conscious effort to find time for their own health and well-being, e.g., through sports, going on walks, or counselling. Carers did not typically consider technology as a solution for dealing with these emotions and finding emotional and social support. One younger carer used social media to vent and explore the humorous side of caring, and to reconnect with friends who had limited understanding of their situation. Several carers noted that there were support groups organized by dementia or carer organizations, but they either conflicted with their work, they did not find the time or energy to attend, or they worried that they did not have much in common with the target audience.

### Safety concerns

All interviewed carers expressed concern for the safety of the person with dementia (Q7). If their work allowed it, many carers used their phone to check in with their care network or the person with dementia—if they did not have difficulties using a phone due to impaired dexterity, hearing, or remembering how to use it. Frequent calls by carers could put a strain on the relationship with the person with dementia, as they could feel patronized by the carer’s attempts to check on them (Q8).

#### Managing accidents and emergencies

All interviewed carers worried about potential accidents like falls or other emergencies in the home. One carer used a system of interconnected sensors to monitor the movement of the person with dementia. Unusual activity could trigger an alert and be monitored via an app or online portal. Despite generally viewing this system positively, the carer noted issues with the interpretation of the transmitted data, particularly if there was more than one person at home. Ian, who worked from home and was a carer simultaneously, suggested a traditional baby phone to hear when his mum needed assistance but did not get one for fear of disturbing her. Cameras were mentioned by many carers, although few had experience using them. Those who did, found cameras with two-way audio transmission, controlled via an app, very useful as it took them mere seconds to check and have peace of mind. Others thought cameras would be too invasive. Many people with dementia were issued community alarm systems by the council, i.e., small wearable panic buttons connected to an emergency response call center via a home base. While some carers were grateful for the technology giving them peace of mind, many others pointed out that people with dementia might not use or forget to use it when needed. Other issues raised included the limited range of the devices, the disembodied voice coming from the home base potentially frightening people with dementia, and the time between setting off the alarm and help arriving. Fall alarms were highly appreciated, as they sent an automatic alarm to the call center when detecting a fall. There were, however, concerns about people with dementia forgetting to wear them, their reliability, and their limited range. One carer suggested an integrated communication function that would make the home base obsolete and increase the range. One person with dementia had been issued a bed occupancy alarm from the council, which would alert the call center if they were out of bed for too long. However, the device produced frequent false alarms, causing the carer many work disruptions and a lot of frustration trying to get the device fixed and diminishing the trust that it would work properly when needed. Furthermore, the carer described the traumatic experience of the person with dementia being woken in the middle of the night by the rescue workers dispatched by the call center (Q9).

#### Reminding

Carers wanted solutions for reminding people with dementia of certain tasks like taking their medication, imminent appointments, and eating and drinking regularly (some also need instructions on how to prepare their food). They also wanted confirmation when such tasks had been completed. One carer found they needed to see what the person with dementia was doing when reminding them to take their medication so they would not take the wrong pills by accident. Regarding automated pill dispensers or recorded messages, carers cautioned that the person with dementia might not hear the notification or hear it but not know what to do about it and get distressed. It was also remarked that pharmacies might refuse to refill automatic dispensers. Carers who worried about people with dementia becoming dehydrated or malnourished wanted technology that could monitor their nutritional and hydration levels. Carers who needed people with dementia to attend appointments on their own wanted solutions to remind them where they needed to be and what they needed to do to get there. Another concern was people with dementia causing fires or floods if they forgot to switch off cookers or faucets.

#### Managing disorientation

Carers worried about people with dementia becoming disorientated and distressed. Some carers found dementia clocks or talking watches helpful. One carer of a person with severe dementia found that video phoning helped soothe them when they were very distressed. A common concern was people with dementia being at risk of getting lost when out for a walk. One person with dementia regularly forgot to pay when shopping, causing their carer to have to disrupt work to sort out the issue with law enforcement. Mobile phones were not a viable solution for people with dementia who did not know how to operate them or forget to bring them along. One carer mentioned a door alarm that would send a notification when the person with dementia leaves home but ended up not using it for fear of frightening them. Many carers suggested GPS tracking devices, but none had any experience with these devices. Some thought the person with dementia might forget to bring the device along. One carer was seemingly so concerned that they half-jokingly suggested implanting their father with a trackable chip.

#### Preventing crime

Carers were concerned about how vulnerable people with dementia were on their own. They specifically mentioned worrying about scammers. Several carers had power of attorney and managed the finances of people with dementia. One carer was so worried that they installed a security camera outside their mother’s home and suggested a doorbell with facial recognition to prevent their mother from admitting strangers.

### Accessing information

#### Finding information

Several carers reported not knowing what support was available and where to turn to for help. Many had not received any information or guidance on dementia, what to expect when caring for a person with dementia, or on caring in general. Others had received information but at a time when they were ill-equipped to understand and process it, found that the information did not fit their needs, or were too exhausted to work through all the information (Q10). Carers wanted easily understandable information about the availability and accessibility of benefits, entitlements, and services including care providers, specialist therapists, day-care-centers, lunch clubs, and technology. Carers expressed wanting practical advice from someone in a similar situation or with experience caring for people with dementia. This could include advice on talking about care-related issues at work, which many found difficult, but which could be essential for accessing support. Carer or dementia organizations or peer groups provided valuable information and guidance but were often difficult to access because their hours conflicted with carers’ work schedules (Q11) and some carers described only learning about them by accident. Carers who used the Internet to look for information sometimes found this overwhelming, as they often did not know what to look for. One carer got information from a social media channel on dementia care and used the comments section to get advice from peers but found that the content was only mildly relevant, as it was from a different country.

#### Fighting for information

For many carers, dealing with the council or organizations involved in aspects of care for people with dementia was cumbersome, bureaucratic, and time-consuming. One frustration was the perceived fragmentation of services. Maggie, for example, described how local councils prescribe a telecare product, technology suppliers provide it, and contractors install and maintain it—with little to no communication between them. This required her to hunt down those responsible for providing a specific aspect of a service, keep calling for updates, and repeat her concerns whenever she was forwarded to someone else. She suggested these organizations could use software to coordinate and work on cases together. Some carers found it very difficult to get the information they needed. While some carers were inclined to excuse councils for being understaffed and overworked, others felt that councils themselves did not know what support was available or how it worked and that obstacles were deliberately put in their path to prevent them from accessing benefits or other forms of support. One carer suggested using a voice recorder app to have a record of the conversations with the council in case they were challenged about what had been agreed.

#### Exchanging information with HCPs

Sometimes people with dementia were able to and preferred to attend appointments with HCPs on their own, in which case carers might worry that they would not get all the relevant new information if they did not attend themselves. Exchanging information with HCPs could be especially challenging due to conflicting work hours. Carers thus suggested a secure online platform where everybody involved in the care of the person with dementia could store and share relevant data such as test results, prescriptions, and care plans.

### Personal care

Many carers provided intense personal care, even if they received help from care services. However, this was only relevant to work–care reconciliation for those who worked and cared at the same place or who worked close enough to care during their lunch breaks. Most personal care (i.e., preparing meals and helping people with dementia to eat, drink, and take their medication; helping them with grooming, etc.) could be planned and arranged around work. Toileting presented a bigger challenge, particularly if people with dementia had incontinence or severely limited mobility, as this could not be planned, took a long time, and was often very uncomfortable for carers. One carer thought about how personal care might be more automated in the future, for example, by robots, but also expressed trepidations regarding artificial intelligence (AI) and robots and believed that the person with dementia would prefer the human touch (Q12).

## Discussion

In this study, we explored which work–care reconciliation challenges working dementia carers needed, wanted, and used technological solutions for. Combining work and care is a dynamic process shaped by fluctuating care needs and changing work demands [26, 38, 44, 52]. The strategies carers use to manage both roles vary according to resources and preferences [52], and it is therefore unsurprising that they reported a wide range of challenges. Carers sought technological support for: (a) care management, (b) attending appointments, (c) entertainment and companionship for the person with dementia, (d) psychological and psychosocial stress, (e) safety concerns, (f) accessing information, and (g) personal care. To preface this discussion, it should be highlighted that while technologies can have a transformative effect on the lives of those who need them, carers and people with dementia alike, they must not be seen as a substitute for sustained investment in health and social care systems [24].

Carers had most experience with technology in relation to safety, whereas few had considered technological support for psychological stress. The interview discussions prompted reflection and creative thinking about potential technological solutions across age groups, echoing previous research [2, 16, 17, 30]. Some needs lend themselves more readily to technological support (e.g., online information or social media peer support), while others, such as companionship or interactions with authorities, are less easily addressed. The technological solutions carers (or, indeed, anyone aiming to support carers via technology) envisage seem to depend greatly on their awareness of existing tools, and this is likely further complicated by rapid technological change and innovation. This article therefore provides a comprehensive account of carers’ needs to support healthcare professionals, researchers, developers, and practitioners in identifying or inferring existing or emerging technologies that may assist in work–care reconciliation. It does not attempt to catalogue available technologies beyond those explicitly mentioned by participants.

Managing the care network was a major challenge. Carers sought tools to coordinate communication, share information, and receive reassurance that agreed care had been delivered. Such solutions (e.g., messaging platforms, shared care records, alerts confirming home visits) depend on the consent and participation of care network members, as well as on care providers procuring software, adapting workflows, training staff, and ensuring data protection compliance. These requirements may be difficult to meet in the current context of social care funding and workforce pressures [15, 59]. Customizable software to support privately organized care was also needed. Secure online platforms were seen as helpful for carers unable to attend appointments, but access again depends on implementation by healthcare providers. Although Covid-19 accelerated remote consultations [31], carers’ participation during work hours and the ability of people with dementia to attend alone require further consideration.

Entertainment and stimulation for people with dementia were a source of concern and guilt for many carers, who worried about the effect of the lack of adequate stimulation and companionship on the progression of symptoms in people with dementia and on their quality of life. Few had considered technological options beyond TV or radio, which were seen as too passive. However, what people with dementia find engaging varies with their personality, abilities, and preferences, which may require carers to reconsider their own assumptions about how their time should be spent and to respect different choices. Suitable technologies thus must account for the individual abilities, preferences, and technology familiarity of people with dementia [37]. Studies indicate potential benefits of tablet-based games [27] and reminiscence apps [41], provided people with dementia can use them independently. Companion robots show promise but raise issues of safety, cost, and acceptability [32, 42, 46]. Similar concerns apply to future technologies such as autonomous vehicles. Communication support technologies must also consider the varied nature of language impairments [61], and carers’ desire for such tools may reflect grief over relational loss rather than communication alone. Accordingly, carers might benefit more from help with accepting and coping with this loss rather than complex technologies.

Safety was an almost universal concern, and the area in which carers most commonly used or wanted technology. They sought systems that detect risk and trigger rapid responses, with flexibility regarding the type of monitoring, who is alerted, and the balance between safety, privacy, and independence. Technologies should be matched to specific risks (e.g., crime, accident, and emergency prevention/response, reminding, disorientation), be responsive to the abilities and preferences of people with dementia (i.e., technology requiring active use or prompting to action vs. passive monitoring technology), provide an adequate range so as not to inadvertently lock people with dementia into a “safe zone,” and present information in easily accessible ways. Technologies aiming to remind or alert people with dementia should also have additional haptic, audio, and/or visual accessibility features. An important issue with technologies that need to be carried or worn by the person with dementia is the potential that they may forget to do so. Devices requiring active use may become unsuitable as dementia progresses [37]. Several carers reported problems with default provision of community alarm systems that were poorly suited to their needs and with fragmented service coordination. For many carers, their need for safety conflicted with the need for privacy and independence of people with dementia. There is, however, some evidence to suggest that technology might improve the independence of people with dementia by increasing their confidence and sense of safety—future research should seek to expand on these findings using more robust methods, including larger sample sizes [39].

For the management of psychological and psychosocial stress and information needs, online resources offer valuable support. Only one carer considered using social media for emotional support, although online peer networks can provide flexible practical and emotional assistance. Psychoeducational programs can improve resilience and reduce stress [6, 33, 35] and should address challenges specific to working carers such as how to talk to line managers and co-workers about their caring responsibilities and how to be assertive when it comes to negotiating autonomy at work—with user involvement in design. Information must be accessible, reliable, up to date, centralized, and certified by trusted sources. System-level digital solutions may also help address fragmented service delivery, although carers have little influence over their implementation.

Personal care needs were generally manageable around work, except toileting and incontinence, which were described as particularly difficult for carers providing care during working hours. Further development in this area is required, and care robots were regarded as unsuitable where personal interaction was preferred.

### Strengths and limitations

To the best of our knowledge, this is the first study to examine which work–care reconciliation challenges dementia carers want, need, or use technological solutions for. In the context of growing pressure on working carers, our findings offer insight into how technology may help them address some challenges and partially compensate for gaps in publicly funded support. A strength of this study is our purposive sampling strategy, which included carers across a wide range of ages, genders, and employment situations. Including carers in their twenties and thirties allowed us to capture the perspectives of a generation for whom digital technologies are a familiar problem-solving resource. Representing diverse employment contexts also contributes to a fuller picture of the challenges carers face and the technologies they may value. However, despite sustained efforts, we were unable to recruit carers in insecure or gig-economy work; future research should explore whether their experiences align with our findings. Although we reached data saturation regarding carers’ challenges and technological needs, and discussed our analysis with other work–care scholars, further engagement with key informants such as carer support organizations could strengthen the credibility of our interpretation.

Future research would benefit from a longitudinal design to examine how carers’ reconciliation strategies evolve over time, how challenges shift across the care trajectory, and whether priorities for technological support change. Ideally, interviews would be conducted at key transition points (e.g., when carers first assume the role, after major health changes for the person with dementia, or following significant changes in employment or care arrangements).

Finally, our findings reflect the context of carers of people with dementia living in Scotland and may not be transferable to other settings.

### Implications for practice

Our findings can support those working with carers (including employers, support organizations, and healthcare professionals) to better understand working dementia carers’ needs in relation to technological solutions. Many carers do not initially consider technology when seeking help, even where promising options exist. Carers should therefore be encouraged to reflect on their work–care challenges and, where other resources are unavailable, be supported in identifying potentially useful technologies. At the same time, technology is not a universal solution, and people with dementia may be unable to use some technologies depending on the progression of their condition. Decisions about technological support should therefore consider both carers’ needs and the implications for the person with dementia.

Several challenges identified in this study would require other stakeholders (such as local authorities, care providers, and healthcare professionals) to change practices and implement supporting technologies, for example, to reduce fragmentation between services. Although this may require initial investment and adaptation, organizations may ultimately benefit from more streamlined workflows and communication with carers. Further research is needed to identify existing technologies that could address the challenges described here. To be genuinely empowering, efforts should aim to support the full range of challenges working dementia carers face, recognizing that many will experience multiple challenges across their working and caring trajectories.

## Conclusion

Dementia carers face many challenges when combining paid work and unpaid care. Technology can offer solutions independent of a strained social care system or of any authorities, thus empowering carers to help themselves. These technologies must be used in a complex context where their impact on people with dementia, on the carers’ work environment, and on everyone involved in the care of people with dementia (i.e., the care network, care services, healthcare providers, local authorities, etc.) must be considered.

## Appendix A

**Table A.1:** Sample quotes for themes and subthemes

Theme	Subtheme	Description	Sample quotes
*Care management*	Coordinating the care network	WDCs need to coordinate responsibilities with their care network	“Do you know what makes the difference? WhatsApp. WhatsApp, because that’s literally a group […] and I will put into that, […] ‘Somebody needs to stop by your gran’s’ or ‘I’ve had to go to [work], can one of you pick up with her,’ and that’s generally it.” [Sue]
		WDCs need to exchange care-related information with their care network	“I just use it [WhatsApp] simply just to, to share, share important notes or it can be anything such as ‘Shall I get some potatoes on the way’ if I’m up here, to communication that relates to an appointment to the doctors.” (Gavin)
		WDCs need reassurance that someone has checked on the PwD	“I always want those [members of the care network] like ‘Guys, talk to me, talk to me,’ […] So yeah, a lot of the time you just haven’t got a clue what’s going on.” (Ian)
	Coordinating care providers	WDCs need to set up and coordinate care services	“The guy who runs the team, I’m in contact with him so frequently, phoning in, emailing him, […] ‘Can you, erm, can you not do this, can you please do that …’.” (Maggie)
		WDCs need to know when care providers arrive at the PwD’s residence so that they can manage work around it	“We’ve no idea when they’re [care workers] gonna be there roughly. No, we know roughly they’re coming between this and this hour. But if you’ve taken your lunch off and then you have to wait for it for ages. […] that’s the kind of, someone, some of that kind of stress that we have if we take our time off and then you want to give her [mum] her lunch and then you end up waiting for ages and then you have stuff to go back at work.” (Max)
		WDCs who privately hire care workers need to manage their responsibilities as employers	“For everything I do there has been an initial time which is gathering the information, understanding what I might need to do, I need to communicate with, particularly in the original set up of the care plan, because I wrote both the job descriptions for the carers, submitted the plan which had to be approved for by the local authority in order that I could make use of the budget that was provided to me.” (Gavin)
*Attending appointments*	Attending medical and similar appointments	WDCs need to attend appointments with HCPs, which is challenging due to conflicting hours	“I’ve had social workers like going ‘Oh yeah we’ll do it before you go to work!’ and you go ‘Well, I leave at 7 o’clock’ and they go ‘Ok wish I’d never said that now!’ So, it tends to be that for those kinds of meetings, the flexibility has to come from my side. […] It’s the same with doctors’ surgeries. You know, they must know that a huge part of the population works.” (Hannah) “The hospital appointments are not so bad because they’re quite far in advance but things like the district nurses, they just turn up on your doorstep and expect you to be there.” (Rose)
	Attending business meetings	WDCs working from home sometimes need to attend business meetings	“Where I think it’s harder is when you’ve got meetings set up with your team, and you’re the person that’s supposed to be going to, I don’t know, some big governance meeting of umpteen people. Where you’re either gonna have to find somebody else to represent the team or you know. You can’t really say to somebody who’s organized something bank-wide, say ‘Look, can you move this cos I need to go and take my mum to the doctor’.” (Hannah)
	Arranging medical or similar appointments	WDCs need to arrange appointments with HCPs, which is challenging due to conflicting hours	“I would like to have access to the GP and the carers through technology. I don’t even have email access to them, so that would be really useful, you know. That’s quite basic technology, but at the moment that all has to be done by phone and for me, actually, email’s easier than phone, especially when you’re working. You can just bang out a quick email rather than having to go and do the whole phone call palaver.” (Theresa)
*Entertainment and companionship*	Providing entertainment and companionship	WDCs want to minimize PwDs’ cognitive decline and social withdrawal	“Sometimes I think that while there is no denying the progress of her disease and the reality of it, sometimes I think that if she could be more stimulated, she wouldn’t have deteriorated so quickly”. (Maggie)
		WDCs want PwD to have more company when they need to work	“I would see her walking past the studio door towards her bedroom and I’d say to her, ‘Oh are you away to bed mum?’ and she’d just look at me and she’d say, ‘What else is there to do,’ you know. And she’s, I know she didn’t mean it to come across like that, but there was an element of, not blame but well, you know, ‘You’re ignoring me, you’re working, what am I going to do, there’s nothing for me to do.’ It wasn’t just, you know, it was, there was a resentment in her voice about it, that she had nothing to do, and she was bored, and she was just going to go to sleep.” (Maggie)
	Enabling active participation in society	WDCs want the PwD to be able to actively participate in society	“If I had a magic wand, I’d just want something to get them out and be happy.” (Jasmin)
	Enabling PwD to communicate	WDCs of PwD whose ability to speak is affected by dementia want to be able to communicate with the PwD	“I’m for the days when people are embedded with a chip, you know, really, I think, that we can communicate telepathically.” (Maggie)
*Psychological and psychosocial stress*	Dealing with psychological and psychosocial stress	WDCs need to deal with complicated emotions, emotional situations, and decisions (e.g., feeling unprepared or abandoned, having to make personal sacrifices, having to manage adverse effects on their health caused by emotional labor and constant vigilance, etc.)	“I’m either feeling guilty about it and I’m not doing my work, guilty about something I’m not doing for my mum or guilty that my kids have had to move down the queue.” (Hannah) “The physical, I couldn’t care less about cutting the grass, picking mum up out of a chair. That’s nothing. (I: So, it’s the emotional labor?), yes, it’s emotionally exhausting.” (Ian)
		WDCs need to deal with interpersonal conflict and difficulties (e.g., role reversal and the slow decline and changing personality of the PwD, difficult relationships between carer and PwD exacerbated by dementia, PwD or their spouse refusing help, etc.)	“It’s like looking after a toddler, but when you are looking after a toddler they are always learning and growing and it’s also a positive beautiful thing. And when you are looking after a toddler like this, they are deteriorating all the time and they are becoming less and less capable and it’s, and to see that happening to your parent [sighs].” (Maggie) “Where to draw a line between being respectful of what she [spouse] wants and saying, ‘You’re being absolutely ridiculous,’ you know? ‘You’re missing out on something here which is detrimental to him because of your views’.” (Sue)
*Safety concerns*	Managing accidents and emergencies	WDCs need reassurance that the PwD is safe (e.g., accidents, falls or other emergencies)	“There’s even the sort of, me sitting at work and I know that my mum’s not well, so I’m maybe not concentrating at much and that has a big effect.” (Flora)
	Reminding	WDCs need to remind PwD of certain tasks or activities (e.g., taking medication, appointments, eating and drinking, etc.) and want confirmation when tasks are completed	“I’m still working full time and as I say if I didn’t have the phone there to check my dad, albeit he gets quite irritated with me checking you know, checking your tablets he’ll go ‘I’m not two’ I’ll go ‘no, but you do forget, so it’s easy for me to pick up the phone and just prompt’.” (Betty)
		WDCs need to remind PwD to switch off appliances which could, e.g., cause fires or floods	“There’ve been quite a few instances where mum’s forgotten she’s put a pan on the stove, um, and they have a system where as soon as the smoke alarm goes off the fire brigade just come.” (Theresa)
	Managing disorientation	WDCs need to ensure PwD remain orientated to avoid distress	“Anything out of his routine, the repercussion’s awful he’s so unsettled afterwards. So, he has a complete fixed routine.” (Sue)
		WDCs need to be able to find PwD who are at risk of getting lost when out for a walk	“He’ll go for wanders, so you lose him, you don’t know where he is and then you’ve got to sort of like try and find out where he is.” (Mary)
	Preventing crime	WDCs need reassurance that PwD are safe from crime (e.g., scammers, burglars, etc.)	“And she’d let this guy into her house, given him £ 100 and then rung me up and said ‘I don’t think I should have done that, should I?’ […] And at that point I did go and look at technology in [department store] where you have some kind of CCTV that links to your phone.” (Hannah)
*Accessing information*	Finding information	WDCs need access to easily understandable information on dementia, caring, benefits, entitlements, and services	“We felt that we’ve been left to fend for ourselves in the sense that we have to do our own investigation and chance conversations, Google searches for things, thinking outside the box, you know.” (Liam)
		WDCs want practical advice from peers or someone with experience caring for PwD	“Sometimes you just need somebody to say well ‘Is this normal?’ you know? Somebody to say, ‘Well is this normal, is this part of the process’ or, you know, ‘Is there something else, you know, sort of going on?’” (Mary)
	Fighting for information	WDCs need reliable information from organizations which can be difficult if more than one organization is involved	“They [council] would assess my mum and then see how much they were prepared to pay for her care. And I was like, ‘How long does that take?’ and she says, ‘Oh, I can’t give you a time,’ and I says, ‘Well, is it an hour, a month or is it six months?’ and she went, ‘Well, it’ll not be six months.’ And that’s what I was left with. And after that, I just kept phoning them and pestering them, which you didn’t want to do because you know that they’re struggling, but every time I phoned, I got a different story. I even said to them, ‘Well how much do you, so that we can work out our finances, how much do you pay an hour?’ and it went from anything from £ 13 to £ 16.” (Flora) “So, you have the council, they run the [telecare] system but they outsource the maintenance to a company called [name], they outsource the supply of the hardware to a different company called [name]. So, these three people, they are all trying to maintain the same system and they don’t talk to each other […]. That [coordinating these services] totally wasted, inefficiency wastes my time, about an hour every day, maybe more, things that don’t need to be.” (Maggie)
	Exchanging information with HCPs	WDCs need to exchange relevant information with HCPs when PwD attend appointments on their own, which is challenging due to conflicting hours	“It’s no good asking my dad for a summary of what happened because you don’t get any information. If they leave information with him, it’s not necessarily gonna get passed on unless I go look for it or ask for it.” (Betty) “I would like to have access to the GP and the carers through technology. I don’t even have email access to them, so that would be really useful, you know. That’s quite basic technology, but at the moment that all has to be done by phone and for me, actually, email’s easier than phone, especially when you’re working. You can just bang out a quick email rather than having to go and do the whole phone call palaver.” (Theresa)
*Personal care*	Toileting/incontinence care	WDCs want solutions for helping PwD to the bathroom, especially incontinence care	“It [incontinence care] is really challenging because it’s your own parent. I mean, it’s bad enough doing that for anybody at all that you are not connected with but to do it for your own parents, it’s such a very difficult and emotional thing.” (Maggie)

*WDCs* working dementia carers, *PwD* person/people living with dementia, *HCPs* healthcare professionals

## Appendix B

### Participant quotes

Q1: “The carers can’t cope, so I have to help them to change her [person with dementia] and do a little personal care. I have to be there to, you know, sometimes there’s new carers […]. So, I have got to be there, to tell them where everything is and how to do everything. And sometimes I get really frustrated and I think I might as well do this myself.” (Maggie) Q2: “It also means on the days that I don’t get to see mum I could just log in and see what they’ve said today […]. I know they would ring me if there was an issue—I know they would ring me—but, sometimes it’s useful just to see a little comment that they’ve put or sometimes they might write that I need to collect more, I need to collect something from the chemist or more nutritional drinks or something like that.” (Theresa) Q3: “For everything I do there has been an initial time which is gathering the information, understanding what I might need to do, I need to communicate with, particularly in the original set up of the care plan, because I wrote both the job descriptions for the carers, submitted the plan which had to be approved for by the local authority in order that I could make use of the budget that was provided to me.” (Gavin) Q4: “You can’t really say to somebody who’s organized something bank-wide,” say, “Look, can you move this ‘cos I need to go and take my mum to the doctor.” (Hannah) Q5: “I would like to have access to the GP and the carers through technology. I don’t even have email access to them, so that would be really useful, you know. That’s quite basic technology, but at the moment that all has to be done by phone and for me, actually, email’s easier than phone, especially when you’re working. You can just bang out a quick email rather than having to go and do the whole phone call palaver.” (Theresa) Q6: “I’m for the days when people are embedded with a chip, you know, really, I think, that we can communicate telepathically.” (Maggie) Q7: “You just have to wait and see what happens every day and deal with every situation. So, you are always firefighting, you can’t plan anything.” (Maggie) Q8: “It’s easy for me to pick up the phone and just prompt, but you know, it’s frustrating for the person who’s forgetting, almost kind of the sense that they’re being told what to do and they’re being monitored. And my dad’s always been a pretty free spirit, so the idea that I’m on his case, checking this, that, and the next thing irritates him.” (Betty) Q9: “These enormous, big blokes come into the house in the middle of the night and wake her up and she’s terrified.” (Maggie) Q10: “That is one of the biggest challenges with this actually, is understanding everything that’s out there and who you need to speak to and what you need to do. And I mean [carer organization] have been good and [dementia organization] have been good but they both sent me piles and piles of leaflets and information and they’re still sitting unread at home because I’ve either been really busy at work or by the time I’ve got home, I’m so exhausted that I don’t want to sit there and read through 15 different leaflets.” (Theresa) Q11: “They’ve [carer organization] got some amazing courses about understanding dementia, you know, how to deal with all the things that are coming. I just can’t get to them because I have work.” (Theresa) Q12: “Maybe in 20 years’ time people are used to that and they find a way of dealing with it. And it’s just because we are on the cusp of that development that we find it frightening. But at the moment, you know, I might say, ‘Well, I would really like it if an automated carer could deal with the incontinence stuff,’ for example. But that’s for my benefit. But mum, how would she feel about that?” (Maggie)

## Footnotes

1 Any quantifiers or semi-quantifiers (e.g., some, many, all, few) used in the following presentation of the findings when referring to participants’ statements only relate to the study sample. These (semi-)quantifiers serve to accentuate patters in the data (i.e., regularities, peculiarities, etc.). No conclusions can be made as to the prevalence of any of the described challenges beyond this study’s population. See Neale et al. [43] for a discussion on the legitimacy and use of (semi-)quantifiers in qualitative research.

## References

[1] Alzheimer’s Research UK (2015). Women and dementia: A marginalised majority. Alzheimer’s Research UK, Cambridge. Available from https://www.alzheimersresearchuk.org/about-us/our-influence/policy-work/reports/women-dementia/

[2] Andersson S, Magnusson L, Hanson E. The use of information and communication technologies to support working carers of older people‑A qualitative secondary analysis. International Journal of Older People Nursing. 2016;11(1):32–43. 10.1111/opn.12087. 26073289 10.1111/opn.12087

[3] Astell A, Smith S, Joddrell P. Using technology in dementia care: A guide to technology solutions for everyday living. London: Jessica Kingsley Publishers; 2019a.

[4] Astell AJ, Bouranis N, Hoey J, Lindauer A, Mihailidis A, Nugent C, Robillard JM. Technology and dementia: The future is now. Dementia and Geriatric Cognitive Disorders. 2019b;47(3):131–9. 10.1159/000497800. 31247624 10.1159/000497800 PMC6643496

[5] Barbour R. Introducing qualitative research: A student’s guide. 2 ed. London: Sage Publications; 2014.

[6] Beauchamp N, Irvine AB, Seeley J, Johnson B. Worksite-based Internet multimedia program for family caregivers of persons with dementia. Gerontologist. 2005;45(6):793–801. 10.1093/geront/45.6.793. 16326661 10.1093/geront/45.6.793

[7] Bergström AL, Hanson E. An integrative review of information and communication technology based support interventions for carers of home dwelling older people. Technology and Disability. 2017;29(1):1–14. 10.3233/TAD-160158. 10.3233/TAD-160158 PMC5814658 29527109

[8] Bourke-Taylor H, Howie L, Law M. Barriers to maternal workforce participation and relationship between paid work and health. Journal of Intellectual Disability Research. 2011;55(5):511–20. 10.1111/j.1365-2788.2011.01407.x. 21385261 10.1111/j.1365-2788.2011.01407.x

[9] Bourke J, Pajo K, Lewis K. Elder care and work-life balance: Exploring the experiences of female small business owners. New Zealand Journal of Employment Relations. 2010;35(1):17–17. http://hdl.handle.net/11072/318.

[10] Braun V, Clarke V. Using thematic analysis in psychology. Qualitative Research in Psychology. 2006;3(2):77–101. 10.1191/1478088706qp063oa.

[11] Broese van Groenou M, De Boer A. Providing informal care in a changing society. Eur J Ageing. 2016;13(3):271–9. 10.1007/s10433-016-0370-7. 27610055 10.1007/s10433-016-0370-7 PMC4992501

[12] Buckner L, Yeandle S (2015) Valuing carers 2015: The rising value of carers’ support. Carers UK, London. Available from https://www.sheffield.ac.uk/polopoly_fs/1.546409!/file/Valuing-Carers-2015.pdf

[13] Bunniss S, Kelly D. Research paradigms in medical education research. Medical Education. 2010;44(4):358–66. 10.1111/j.1365-2923.2009.03611.x. 20444071 10.1111/j.1365-2923.2009.03611.x

[14] Calvano L. Tug of war: Caring for our elders while remaining productive at work. The Academy of Management Perspectives. 2013;27(3):204–18. 10.5465/amp.2012.0095.

[15] Care Quality Commission. The state of health care and adult social care in England 2020/21. 2021. https://www.cqc.org.uk/sites/default/files/20211021_stateofcare2021_print.pdf.

[16] Carers UK (2012). Carers and telecare. Carers UK, London. Available from https://www.carersuk.org/for-professionals/policy/policy-library/carers-and-telecare-report

[17] Carers UK. Potential for change: transforming public awareness and demand for health and care technology. 2013a. https://www.carersuk.org/for-professionals/policy/policy-library/potential-for-change-transforming-public-awareness-and-demand-for-health-and-care-technology.

[18] Carers UK (2013b). State of caring survey 2013. Carers UK, London. Available from www.carersuk.org/for-professionals/policy/policy-library/the-state-of-caring-2013

[19] Carers UK (2014). Supporting employees who are caring for someone with dementia. Carers UK, London. Available from https://www.carersuk.org/for-professionals/policy/policy-library/supporting-employees-who-are-caring-for-someone-with-dementia

[20] Carers UK. Juggling work and unpaid care: A growing issue. Carers UK, London. 2019. http://www.carersuk.org/images/News_and_campaigns/Juggling_work_and_unpaid_care_report_final_0119_WEB.pdf.

[21] Carers UK. Carers Week 2020 Research Report: The rise in the number of unpaid carers during the coronavirus (COVID-19) outbreak. 2020. https://www.carersweek.org/images/CW%202020%20Research%20Report%20WEB.pdf.

[22] Centers for Disease Control and Prevention. Promoting caregiving across the full community: A public health strategy (pp. 1–66). U.S. Department of Health and Human Services. 2020. https://www.cdc.gov/caregiving/media/pdfs/promoting-caregiving-across-the-full-community-202012-5081.pdf.

[23] Charles A, Ewbank L (2021). The road to renewal: Five priorities for health and care. The King’s Fund, London. Available from https://www.kingsfund.org.uk/publications/covid-19-road-renewal-health-and-care#now

[24] Eccles A. Remote care technologies, older people and the social care crisis in the United Kingdom: a Multiple Streams Approach to understanding the ‘silver bullet’of telecare policy. Ageing Soc. 2021;41(8):1726–47. 10.1017/S0144686X19001776.

[25] Eldh AC, Carlsson E. Seeking a balance between employment and the care of an ageing parent. Scandinavian Journal of Caring Sciences. 2011;25(2):285–93. 10.1111/j.1471-6712.2010.00824.x. 20723154 10.1111/j.1471-6712.2010.00824.x

[26] Gallagher-Thompson D, Choryan Bilbrey A, Apesoa-Varano EC, Ghatak R, Kim KK, Cothran F. Conceptual framework to guide intervention research across the trajectory of dementia caregiving. The Gerontologist. 2020;60(S1):29–40. 10.1093/geront/gnz157. 10.1093/geront/gnz157 PMC7019661 32057080

[27] Groenewoud H, De Lange J, Schikhof Y, Astell A, Joddrell P, Goumans M. People with dementia playing casual games on a tablet. Gerontechnology. 2017;16(1):37–47.

[28] Hamblin KA, Hoff A (2012). Carers@work: interviews with working carers: summary report. University of Oxford. Oxford Institute of Population Ageing, Oxford. Available from http://www.ageing.ox.ac.uk/download/57

[29] Huang SS, Lee MC, Liao YC, Wang WF, Lai TJ. Caregiver burden associated with behavioral and psychological symptoms of dementia (BPSD) in Taiwanese elderly. Arch Gerontol Geriatr Plus. 2012;55(1):55–9. 10.1016/j.archger.2011.04.009. 21601931 10.1016/j.archger.2011.04.009

[30] Jarrold K, Yeandle S. A weight off my mind’: Exploring the impact and potential benefits of telecare for unpaid carers in Scotland. Carers Scotland, Glasgow. 2009. https://essl.leeds.ac.uk/download/downloads/id/627/carers_scotland_report_december_2009.pdf.

[31] Joy M, et al. Reorganisation of primary care for older adults during COVID-19: A cross-sectional database study in the UK. British Journal of General Practice. 2020;70(697)e540. 10.3399/bjgp20X710933. 10.3399/bjgp20X710933 PMC7363277 32661009

[32] Jung MM, van der Leij L, Kelders SM. An exploration of the benefits of an animallike robot companion with more advanced touch interaction capabilities for dementia care. Front ICT. 2017;4:16. 10.3389/fict.2017.00016.

[33] Klemm PR, Hayes ER, Diefenbeck CA, Milcarek B. Online support for employed informal caregivers. CIN: Computers, Informatics, Nursing. 2014;32(1):10–20. 10.1097/CIN.0000000000000009. 24284908 10.1097/CIN.0000000000000009

[34] Koyama A, Matsushita M, Hashimoto M, Fujise N, Ishikawa T, Tanaka H, Hatada Y, Miyagawa Y, Hotta M, Ikeda M. Mental health among younger and older caregivers of dementia patients. Psychogeriatrics. 2017;17(2):108–14. 10.1111/psyg.12200. 26968528 10.1111/psyg.12200

[35] Kuhn D, Hollinger-Smith L, Presser J, Civian J, Batsch N. Powerful Tools for Caregivers Online: an innovative approach to support employees journal of workplace. J Workplace Behav Health. 2008;23(1-2):51–69. 10.1080/15555240802188630.

[36] Kuo LM, Huang HL, Hsu WC, Shyu YI. Health-related quality of life and self-efficacy of managing behavior problems for family caregivers of vascular dementia and Alzheimer’s disease patients. Dementia and Geriatric Cognitive Disorders. 2014;38(5):310–20. 10.1159/000360414. 25011490 10.1159/000360414

[37] Lorenz K, Freddolino PP, Comas-Herrera A, Knapp M, Damant J. Technology-based tools and services for people with dementia and carers: Mapping technology onto the dementia care pathway. Dementia. 2019;18(2):725–41. 10.1177/1471301217691617. 28178858 10.1177/1471301217691617

[38] Matsumoto N, Ikeda M, Fukuhara R, Shinagawa S, Ishikawa T, Mori T, Toyota Y, Matsumoto T, Adachi H, Hirono N. Caregiver burden associated with behavioral and psychological symptoms of dementia in elderly people in the local community. Dementia and Geriatric Cognitive Disorders. 2007;23(4):219–24. 10.1159/000099472. 17299264 10.1159/000099472

[39] Meiland F, et al. Technologies to support community-dwelling persons with dementia: a position paper on issues regarding development, usability, effectiveness and cost-effectiveness, deployment, and ethics. JMIR Rehabil Assist Technol. 2017;4:e1-e1. 10.2196/rehab.6376. 28582262 10.2196/rehab.6376 PMC5454557

[40] Moon H, Dilworth-Anderson P. Baby boomer caregiver and dementia caregiving: findings from the National Study of Caregiving. Age Ageing. 2015;44(2):300–6. 10.1093/ageing/afu119. 25359299 10.1093/ageing/afu119 PMC4339725

[41] Moon S, Park K. The effect of digital reminiscence therapy on people with dementia: a pilot randomized controlled trial. BMC Geriatr. 2020;20(1)166. 10.1186/s12877-020-01563-2. 32375661 10.1186/s12877-020-01563-2 PMC7204054

[42] Moyle W, Jones C, Sung B, Bramble M, O’Dwyer S, Blumenstein M, Estivill-Castro V. What effect does an animal robot called CuDDler have on the engagement and emotional response of older people with dementia? A pilot feasibility study. Int J Soc Robot. 2016;8:145–56. 10.1007/s12369-015-0326-7.

[43] Neale J, Miller P, West R. Reporting quantitative information in qualitative research: Guidance for authors and reviewers. Addiction. 2014;109(2):175–6. 10.1111/add.12408. 24422609 10.1111/add.12408

[44] Newbronner L, Chamberlain R, Borthwick R, Baxter M, Glendinning C (2013). A road less rocky: Supporting carers of people with dementia. Carers Trust, London. Available from A road less rocky: Supporting carers of people with dementia

[45] Phillips D, Paul G, Fahy M, Dowling-Hetherington L, Kroll T, Moloney B, Duffy C, Fealy G, Lafferty A. The invisible workforce during the COVID-19 pandemic: Family carers at the frontline. HRB Open Res. 2020;3:24–24. 10.12688/hrbopenres.13059.1. 32551415 10.12688/hrbopenres.13059.1 PMC7276936

[46] Pike J, Picking R, Cunningham S. Robot companion cats for people at home with dementia: A qualitative case study on companotics. Dementia. 2021;20(4):1300–18. 10.1177/1471301220932780. 32668978 10.1177/1471301220932780

[47] Prince M, Comas-Herrera A, Knapp M, Guerchet M, Karagiannidou M (2016). World Alzheimer report 2016: improving healthcare for people living with dementia: coverage, quality and costs now and in the future. Alzheimer’s Disease International, London. Available from https://www.alzint.org/u/WorldAlzheimerReport2016.pdf

[48] Prince M, Knapp M, Guerchet M, McCrone P, Prina M, Comas-Herrera A, Wittenberg R, Adelaja B, Hu B, King D, Rehill A, Salimkumar D. Dementia UK: Update. 2014. https://www.alzheimers.org.uk/sites/default/files/migrate/downloads/dementia_uk_update.pdf?fileID=2323.

[49] Round A (2017). Extending working lives: A devolved, lifecourse approach to enabling work beyond state pension age. Institute for Public Policy Research, UK. Available from https://www.ippr.org/publications/extending-working-lives

[50] Smith C. Technology and web-based support. Journal of Social Work Education. 2008;44(S3):75–82. 10.5175/JSWE.2008.773247715.

[51] Spann A, Vicente J, Abdi S, Hawley M, Spreeuwenberg M, de Witte L. Benefits and barriers of technologies supporting working carers—A scoping review. Health & Social Care in the Community. 2022;30:e1–e15. 10.1111/hsc.13421. 34036665 10.1111/hsc.13421

[52] Spann A, Vicente J, Allard C, Hawley M, Spreeuwenberg M, de Witte L. Challenges of combining work and unpaid care, and solutions: A scoping review. Health & Social Care in the Community. 2020;28(3):699–715. 10.1111/hsc.12912. 31845451 10.1111/hsc.12912

[53] Sriram V, Jenkinson C, Peters M. Informal carers’ experience of assistive technology use in dementia care at home: a systematic review. BMC Geriatr. 2019;19(1)160. 10.1186/s12877-019-1169-0. 31196003 10.1186/s12877-019-1169-0 PMC6567448

[54] Utz RL, Lund DA, Caserta MS, Wright SD. The benefits of respite time-use: A comparison of employed and nonemployed caregivers. J Appl Gerontol. 2012;31(3):438–61. 10.1177/0733464810389607.

[55] Waite L, Grayson D, Jorm AF, Creasey H, Cullen J, Bennett H, Casey B, Broe GA. Informant-based staging of dementia using the clinical dementia rating. Alzheimer’s Disease & Associated Disorders. 1999;13(1):34–7. 10.1097/00002093-199903000-00005. 10.1097/00002093-199903000-00005 10192640

[56] Wang YN, Hsu WC, Shyu YL. Job demands and the effects on quality of life of employed family caregivers of older adults with dementia: a cross-sectional study. Journal of Nursing Research. 2020;28(4)e99. 10.1097/jnr.0000000000000383. 10.1097/jnr.0000000000000383 32501963

[57] Wang YN, Shyu YI, Tsai WC, Yang PS, Yao G. Exploring conflict between caregiving and work for caregivers of elders with dementia: a cross-sectional, correlational study. Journal of Advanced Nursing. 2013;69(5):1051–62. 10.1111/j.1365-2648.2012.06092.x. 22776026 10.1111/j.1365-2648.2012.06092.x

[58] Wang YN, Shyu YIL, Chen MC, Yang PS. Reconciling work and family caregiving among adult-child family caregivers of older people with dementia: Effects on role strain and depressive symptoms. Journal of Advanced Nursing. 2011;67(4):829–40. 10.1111/j.1365-2648.2010.05505.x. 21077933 10.1111/j.1365-2648.2010.05505.x

[59] Ward L, Ray M, Tanner D. Understanding the social care crisis in England through older people’s lived experiences. In: Urban P, Ward L, editors. Care ethics, democratic citizenship and the state. Cham: Springer International Publishing; 2020. pp. 219–39. 10.1007/978-3-030-41437-5_11.

[60] Wasilewski MB, Stinson JN, Cameron JI. Web-based health interventions for family caregivers of elderly individuals: a scoping review. Int J Med Inform. 2017;103:109–38. 10.1016/j.ijmedinf.2017.04.009. 28550996 10.1016/j.ijmedinf.2017.04.009

[61] Weekes BSH. Aphasia in Alzheimer’s disease and other dementias (ADOD): evidence from Chinese American. Journal of Alzheimer’s Disease Other Dementias. 2020;35:1533317520949708. 10.1177/1533317520949708. 10.1177/1533317520949708 PMC10624002 33040568

[62] Wittenberg R, Hu B, Barraza-Araiza L, Rehill A (2019). Projections of older people living with dementia and costs of dementia care in the United Kingdom 2019-2040. CPEC Working Paper 5. London School of Economics and Political Science, London. Available from https://www.alzheimers.org.uk/sites/default/files/2019-11/cpec_report_november_2019.pdf

[63] Witzel A. The problem-centered interview. Forum Qualitative Sozialforschung / Forum: Qualitative Social Research. 2000; 10.17169/fqs-1.1.1132.

[64] World Health Organization. Global action plan on the public health response to dementia 2017–2025. Geneva, Switzerland: World Health Organization; 2017.

[65] Yeandle S, Buckner L. Carers, employment and services: Time for a new social contract? 2007. http://circle.group.shef.ac.uk/wp-content/uploads/2018/04/CES-6-EWS4031Time-for-a-new-social-contract.pdf.

[66] Yellowlees R (2020). Dementia and Technology: A literature review and qualitative study. The Life Changes Trust, Glasgow. Available from https://www.lifechangestrust.org.uk/sites/default/files/Dementia%20and%20Technology%20-%20A%20Literature%20Review%20and%20Qualitative%20Study%20report_0.pdf.

